# 

**Published:** 1893-02-11

**Authors:** 


					1893. THE HOSPITAL.
?ciooirt,i_,?, . CONTENTS.
,an.d Work
cor
307
&t0^fth!i'?ood and
?^cai fe ?0sPitals
303
?. *lTBrW?A8*?*y frOE
C^^sero* wy. S?!* ?*? Earliest Times.
By E. T.
XLII.?The Mediaeval Physician
309
?iJ~Polonr and Research.-
-Engenolacetic Acid
The Health of India-
-Why do we Shiver ?
SIO
CESSS
By Mrs. Ebnest Habt.
XII.?Diabetes ?,
fio?nf?e^se*~By Mr
Clinic-
The Xiex CUniC-
?^featniBT,?^????SPITAL F0n Disease op the
Hip.-
-The
315
??Ui, HosVtS Morbna Coxae
Sick Chi
,?ln Siok Ohii^T F05.. ICK Childben, Glasgow..
-Spinal Caries
m Sick nwM F02.Sick Childeen, Glabgc
?5 ?nd Treatment
816
PnAi^T, ren' DiaKnosi? and Tr
w*attuaa]S^^!- Bookshelf..
?Embryology of Man ana
v*aO?na]?ii- 8 Bookshexf. ?Embrj
N>W DiSS~K?dlt??i'eaii ~
. Winter Reaorta-
318
Dttuoq 7*f<3lterraBean Winter Resorts-
us? Appliances, and Things Medical-
Safety Lamps
818
Everybody's Pago
312
Annotations.
- Experimental Anti-choleraic Inoculations?The
Food of #< Poor Jack ?Mr. J. H. Hewer, M.E.O.S.?
313
XTotes and Sews ?
The Institutional Workshop?
Within the Hospitals -
-Sunderland Infirmary-
319
Hospital Construction.-
-Cottage Hospital. Keswick,
319 ;
General Oiril Hospital, Colombo, Oeylon
320
Asylums nr India.
-The Madras rresiaency
SJO
The Stoey of the Insane feom Yeae to Yeab
321
Hospital Opinion.-
-Bedford and its Medical Institutions?
Bedfordshire Hospital Trained Norses' Institute
321
Construction Notes-
?Another Hint to the Oxford Infirmary
Scraps and Gleanlnffs -
The Student of Medicine.?Appointments?Vacancies.
tmr*. ^ extra supplement.?the nursing mirror.
*6?? from EXTRA SUPPIEME1
Nursirg- World.?Little Invalids?The
I*oor~-TT?,_. ?-,"'easant Entertainment?The Bonrnemonth
DundoU!^? i?1 Death-Beds?Mrs. or Miss f?Progress
?uraea' ti^elbonrne Nurses?Nurses at Varcouver?
l, Item8__?50nie, Boston, U.S.A.? Native Whims ?Short
aaageinpi+e ,CBP'tal" Endowed Bed?A Disabled Nnrse
?nv tion of nil of Consumptives. I.?General Desorip
UtiraeB^Ption
?ia,lliaatio?^00kshelf
Question for March. - - - -
Ti^riilrm Obstetric Society -
czli
czliii
cxliii
czliii
cxliv
The Nurses' co-operation
Everybody's Opinion.?Indian Nursing?America Diets?
Wanted, G-ood Nnrses?Girl Nurses and Infirmaries?Mrs. "Ward-
roner?A Disabled Nurse?" The Hospital" Etdowed Bed cxlv
The International Nursing- Congress, Chicago - czlv
Croupous Pneumonia cxlvi
Requisites for Nurses, cxliv; Notes and Queries- ? czlyi
Por Beading to the Sick.?Training cxlyii
The History of Nursing cxlvii
Some Australian Experiences.?My Reception ? - cxlviii
Presentations cxlviii

				

